# Autophagy and SARS-CoV-2 infection: A possible smart targeting of the autophagy pathway

**DOI:** 10.1080/21505594.2020.1780088

**Published:** 2020-06-22

**Authors:** Shahla Shojaei, Madhumita Suresh, Daniel J. Klionsky, Hagar Ibrahim Labouta, Saeid Ghavami

**Affiliations:** a College of Pharmacy, Rady Faculty of Health Sciences, University of Manitoba, Winnipeg, Manitoba, Canada; b Life Sciences Institute and Department of Molecular, Cellular and Developmental Biology, University of Michigan, Ann Arbor, Michigan, USA; c Children’s Hospital Research Institute of Manitoba, Winnipeg, Manitoba, Canada; d Biomedical Engineering Program, University of Manitoba, Winnipeg, Manitoba, Canada; e Department of Human Anatomy and Cell Science, Max Rady College of Medicine, Rady Faculty of Health Sciences, University of Manitoba, Winnipeg, Manitoba, Canada; f Department of Pharmaceutics, Faculty of Pharmacy, Alexandria University, Alexandria, Egypt; f Research Institute in Oncology and Hematology, CancerCare Manitoba, University of Manitoba, Winnipeg, Manitoba, Canada; g Autophagy Research Centre, Health Policy Research Center, Institute of Health, Shiraz University of Medical Sciences, Shiraz, Iran; h Faculty of Medicine, Katowice School of Technology, Katowice, Poland

**Keywords:** Apoptosis, autophagy flux, drug targeting, macroautophagy, nanomedicine, nanoparticles, SARS-CoV-2

## Abstract

The severe acute respiratory syndrome coronavirus 2 (SARS-CoV-2) outbreak resulted in 5,993,317 confirmed cases worldwide with 365,394 confirmed deaths (as of May 29^th^, 2020, WHO). The molecular mechanism of virus infection and spread in the body is not yet disclosed, but studies on other betacoronaviruses show that, upon cell infection, these viruses inhibit macroautophagy/autophagy flux and cause the accumulation of autophagosomes. No drug has yet been approved for the treatment of SARS-CoV-2 infection; however, preclinical investigations suggested repurposing of several FDA-approved drugs for clinical trials. Half of these drugs are modulators of the autophagy pathway. Unexpectedly, instead of acting by directly antagonizing the effects of viruses, these drugs appear to function by suppressing autophagy flux. Based on the established cross-talk between autophagy and apoptosis, we speculate that over-accumulation of autophagosomes activates an apoptotic pathway that results in apoptotic death of the infected cells and disrupts the virus replication cycle. However, administration of the suggested drugs are associated with severe adverse effects due to their off-target accumulation. Nanoparticle targeting of autophagy at the sites of interest could be a powerful tool to efficiently overcome SARS-CoV-2 infection while avoiding the common adverse effects of these drugs.

Viruses recruit cellular machinery and pathways, such as autophagy, for their replication and spread [1, 2]. Autophagy is a part of the cell stress response that works as a quality control mechanism for cells by removing and degrading malfunctioning proteins, damaged organelles, and invasive microbes [1,3]. Macroautophagy, hereafter autophagy, is initiated via the formation of a double-membrane structure (termed a phagophore). The phagophore engulfs the substrates that are targeted for ultimate degradation, and sequesters them within an autophagosome. The mature autophagosome merges with a lysosome to generate an autolysosome where the engulfed material will be degraded [1,4].

Hijacking of cellular autophagy mechanisms has been reported for several viruses. For example, measles virus/MeV induces autophagy through the engagement of CD46; human immunodeficiency virus type 1/HIV-1 envelope glycoproteins gp120 and gp41 induce autophagy in uninfected CD4^+^ T cells and initiate HIV-1 entry with subsequent T cell apoptosis and immunodeficiency; Chikungunya virus/CHIKV triggers autophagy via an endoplasmic reticulum and oxidative stress pathway [5]; Macacine alphaherpesvirus 1/MCHV, and murine gammaherpesvirus (MHV) 68/MHV-68 inhibit autophagy by blocking phagophore formation [5]; Picornaviruses, coxsackie virus and coronaviruses utilize autophagy to promote their replication [5]. Although these viruses hijack cellular autophagy pathways in favor of their replication and transcription, for other viruses autophagy restricts the viral infection by degrading engulfed viruses in a process called virophagy [5].

The SARS-CoV-2 global outbreak, responsible for coronavirus disease 2019 (COVID-19) [6,7], belongs to the betacoronavirus (βCoV) genus. This genus also includes SARS-CoV, Middle East respiratory syndrome-coronavirus (MERS-CoV) and MHV [8]. βCoV are positive-sense RNA viruses [9]. Among them, MHV has been used as a prototype for βCoV in biological investigations. βCoV utilize double-membrane vesicles (DMVs), which are similar to autophagosomes, for their replication [10]. Using MHV-infected delayed brain tumor/DBT cells, Prentice, and co-workers were the first to show the replication of βCoV inside DMVs [11]. They also showed that βCoV induce ATG5-dependent autophagy [11]. Another study confirmed βCoV induction of ATG5-dependent autophagosome formation via their NSP6 (non-structural protein 6) in MHV-infected VERO cells [12]. Similarly, viral membrane-anchored papain-like protease/PLpro-TM polyprotein produced by both SARS-CoV and MERS-CoV induces the formation of autophagosomes, but inhibits their maturation, preventing the generation of autolysosomes as shown in three different human cell lines [13]. In line with these reports, a recent study, using *ATG5* wild-type and *ATG5* knockout Vero B4 cells, reported that MERS-CoV infection suppresses autophagy flux by inhibiting the fusion step [14]. In contrast, few studies reported a βCoV infection which is independent of autophagy induction mechanisms [15,16]. For example, Reggiori and co-workers confirmed that replication and release of βCoV are independent of autophagy [15]. However, they showed that the virus utilizes DMVs coated with non-lipidated microtubule-associated protein 1 light chain 3 (LC3)-I for replication. To the best of our knowledge, no similar experiments have been conducted using SARS-CoV-2. However, an evolutionary analysis on SARS-CoV-2 genome sequences of 351 clinical samples revealed mutations in NSP6, a protein that has an inducing effect on autophagosome formation [17]. This finding infers an interaction of SARS-CoV-2 cell infection and autophagy (Figure 1).

**Figure 1.**
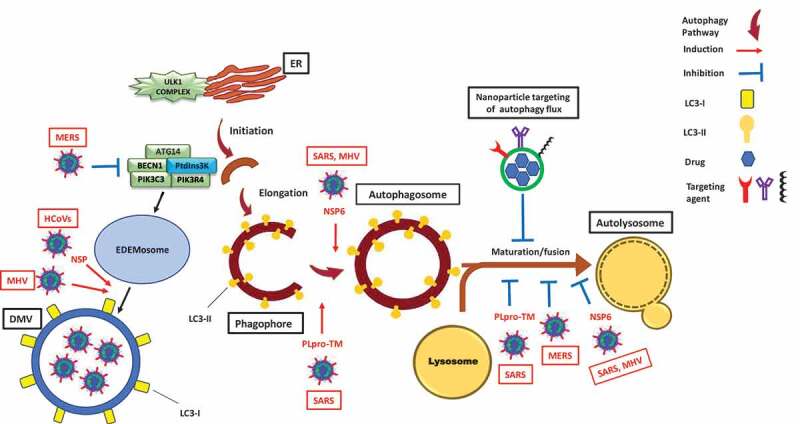
Modulation of the autophagy pathway by coronaviruses and proposal of novel smart drug-loaded nanoparticles to target this pathway to combat COVID-19. Schematic shows how coronaviruses interact with autophagy. The NSP6 protein of SARS and MHV induces the formation of autophagosomes but confines their expansion and blocks their maturation into autolysosomes. A similar effect is observed by PLpro-TM of SARS. Human CoVs (HCoVs) via their NSPs, and MHV induce the formation of LC3-I-coated DMVs needed for viral RNA transcription and replication. MERS decreases the level of BECN1 (beclin 1) and blocks fusion of autophagosomes with lysosomes. Chloroquine/hydroxychloroquine, emtricitabine/tenofovir, interferon alfa-2b, lopinavir/ritonavir and ruxolitinib, which are all under clinical trial for treatment of SARS-CoV-2, induce autophagosome accumulation by blocking their maturation into autolysosomes. Thus, designing nanoparticles for the targeted delivery of these drug to avoid their off-target effects will provide safe and effective powerful tools to combat COVID-19. ATG14: autophagy related 14; DMV: double-membrane vesicles; EDEMosome: LC3-I-positive endoplasmic reticulum-derived vesicles exporting short-lived ERAD regulators; ER: endoplasmic reticulum; LC3-I: processed MAP1LC3; LC3-II: lipidated MAP1LC3; MERS: Middle East respiratory syndrome; MHV: murine gammaherpes virus; NSP6: non-structural protein 6; PIK3C3/VPS34: phosphatidylinositol 3-kinase catalytic subunit type 3; PIK3R4/VPS15: phosphoinositide-3-kinase regulatory subunit 4; PtdIns3 K: class III phosphatidylinositol 3-kinase; PLpro-TM: membrane-anchored papain-like protease; SARS: severe acute respiratory syndrome; ULK1 complex: unc-51 like autophagy activating kinase 1.

COVID-19 is associated with common symptoms such as fever and shortness of breath. These symptoms could progress to an acute respiratory distress syndrome/ARDS that leads to lung failure, the most common reason of death [18]. To date, there is no clinically approved drug to prevent or cure COVID-19. Repurposing of FDA-approved drugs was associated with promising outcomes and resulted in ongoing clinical trials for 12 drugs tested against COVID-19, based on a recent WHO report [19]. Several potential drug candidates are autophagy modulators (Table 1). Surprisingly, almost all of these autophagy modulators do not appear to act by directly antagonizing the effect of βCoVs. Instead, they inhibit autophagy flux in a similar fashion to the effect of βCoVs (Figure 1). Therefore, we suggest that *the beneficial effect of these drugs is possibly due to the over-accumulation of autophagosomes that can potentially induce apoptotic cell death of virally infected cells and disrupt the virus replication cycle, similar to what we observed in our recent study* [20].

**Table 1.** Drugs under clinical trials against SARS-CoV-2 infection based on the World Health Organization report [19], their autophagy-related mechanism of action, and their severe side-effects.

Drug Name	Autophagy-related mechanism of action	Side effects
CQ/HCQ	Inhibits autophagy flux by decreasing autophagosome-lysosome fusion [40]	Retinopathy, gastrointestinal effects, cardiomyopathy, myopathy [41]
Corticosteroids	Inhibits autophagy by blocking LC3 recruitment [42]	Myopathy, osteopenia/osteoporosis, decreased sex hormones [43]
Emtricitabine/Tenofovir	Increases expression and accumulation of SQSTM1/p62 [44], decreases fusion of autophagosomes with lysosomes [45]	Renal toxicity [46]
Interferon alfa-2b	Induces autophagy and accumulation of autolysosomes [47]	Flu-like symptoms, nausea, anorexia, depression, confusion, myalgia, fatigue, joint pain [25] retinopathy, neuropsychopathy [48]
Lopinavir/Ritonavir	Induces autophagosome accumulation [49]	Gastrointestinal effects, headache, diabetes, hyperbilirubinemia, dizziness [50]
Ruxolitinib	Downregulates the MTORC1-RPS6KB-EIF4EBP1 pathway [51], induces accumulation of autophagosomes [52]	Anemia, pancytopenia [53]

EIF4EBP1: eukaryotic translation initiation factor 4E binding protein I; LC3: microtubule-associated protein 1 light chain 3; MTOR: mechanistic target of rapamycin kinase; RPS6KB/p70S6K: ribosomal protein S6 kinase B; SQSTM1/p62: sequestosome 1

It is very important to consider the unfolded protein response (UPR), an important intracellular pathway that is activated as a response to the accumulation of unfolded proteins in the endoplasmic reticulum (ER) with regard to viral infection. The UPR is usually activated during coronavirus infection because virus replication requires excessive protein biosynthesis and folding to provide sources for viral proteins, and use of the ER membrane for the formation of DMVs [21,22]. Furthermore, the UPR and autophagy are interconnected, and induction of the UPR could potentially facilitate or promote autophagy [4,23,24]. Therefore, SARS-CoV-2 infection could possibly induce autophagy via UPR induction in the cells.

As depicted in Table 1, all of the indicated drugs have severe adverse effects and limited patient tolerance. This is attributed to the off-target effects of these drugs upon systemic administration [25]. For instance, chloroquine/CQ has some potential as an effective therapy for COVID-19 based on preliminary clinical trial findings [26], but is associated with retinopathy, neuromyopathy, nephropathy, and cardiomyopathy that makes it difficult to tolerate [27,28].

The body of literature pointing to the mutual effect of SARS-CoV-2 infection and autophagy, in addition to the fact that 58% of the drugs under clinical trials for COVID-19 are autophagy modulators [26], emphasize the need for research in the area of autophagy for the fight against COVID-19. It is very important to consider that the drugs in Table 1 modulate other mechanisms than auto-phagy to decrease SARS-CoV-2 infection. As an example, chloroquine/hydroxychloroquine has anti-inflammatory effects and might be involved in controlling a SARS-CoV-2-induced cytokine storm [29], endocytosis of the virus [30], and regulation of the SARS-COV-2 receptor, ACE2 (angiotensin I converting enzyme 2) [29]. Some of these effects, including regulation of the cytokine storm, and endocytosis of the virus are indirectly regulated by auto-phagy [30].

Therefore, we recommend two main research targets for scientists who are investigating the interconnection of viral infection and autophagy:

- Mechanistic understanding of the intracellular trafficking and replication of SARS-CoV-2.
- Developing effective therapies that are specific to SARS-CoV-2 and the autophagy pathway.

Successful implementation of an autophagy modulator as a safe and efficacious therapy for COVID-19 requires a carrier to deliver it to the site of action (infected cells) and mitigate off-target effects. Applications of nanotechnology in medicine (called nanomedicine), have introduced the use of nanoparticles for targeting active sites and avoiding off-target accumulation. This is based on the unique physical properties of nanoparticles, which affect their bioavailability and circulation time. Decorating the nanoparticles with ligands directed to specific cell targets amplifies nanoparticle specificity [31,32]. Other advantages offered by nanoparticles include their ability to cross biological barriers [33], improved bioavailability of poorly soluble drugs (based on the large surface-area-to-volume ratio of nanoparticles compared to large particles) [34] and tunability of nanoparticle surface charge and chemistry to further control interactions with cells and barriers [33,35]. Recently, nanoparticles were shown to modulate auto-phagy, and have been exploited for overcoming obstacles encountered with autophagy modulators [36]. Several nanoparticle-based products are approved or under evaluation for the treatment of viral infections, including Inflexal V® (Crucell, Berna Biotech), and PegIntron® (Merck) [37]. Therefore, nanotechnology has a great potential for contributing significantly to the fight against COVID-19 by developing effective therapies that can selectively block the replication of the virus in target cells [38].

Further, SARS-CoV-2 could be considered as natural spherical nanoparticles (60- to 140-nm size range). Therefore, mechanisms established for nanoparticle interaction with target cells and subcellular organelles, could be used to enhance our understanding of cell binding and intracellular trafficking mechanisms of the virus [39]. *We strongly recommend cross-disciplinary collaborations between autophagy and nanotechnology communities in order to accelerate the discovery of potential drug candidates and the translation of these discoveries into clinically-approved COVID-19 therapies that are both effective and safe.*

## Figures and Tables

**Table:** 

ATG5	autophagy related 5
βCoV	betacoronavirus
DMVs	double-membrane vesicles
HIV-1	human immunodeficiency virus - I
MAP1LC3/LC3	microtubule associated protein 1 light chain 3
MERS-CoV	Middle East respiratory syndrome-coronavirus
MHV	murine gammaherpesvirus
NSP6	non-structural protein 6
SARS-CoV	severe acute respiratory syndrome-coronavirus
SARS-CoV-2	severe acute respiratory syndrome-coronavirus 2
UPR	unfolded protein response
WHO	World Health Organization
